# Analysis of key obstacles affecting long-term inhalation therapy compliance in children with bronchial asthma at primary hospitals

**DOI:** 10.3389/fped.2026.1721779

**Published:** 2026-02-12

**Authors:** Dianbiao Fan, Yuejin Wu, Wenjing Shi

**Affiliations:** 1 Department of Pediatrics, Jinshan Branch of Shanghai Sixth People’s Hospital (Jinshan Central Hospital Affiliated to Shanghai University of Medicine & Health Sciences), Shanghai, China; 2 Department of Pediatrics, Shanghai Sixth People’s Hospital Affiliated to Shanghai JiaoTong University of Medicine, Shanghai, China

**Keywords:** asthma, child, inhalation, medication adherence, primary health care

## Abstract

**Background:**

Bronchial asthma is a prevalent chronic respiratory disease in children, with long-term inhalation therapy being essential for disease control; however, adherence remains a significant challenge, particularly in primary care settings. This study aimed to identify the key obstacles affecting long-term inhalation therapy compliance among pediatric asthma patients managed at primary hospitals.

**Methods:**

A retrospective analysis was conducted on 268 children aged 4–16 years with bronchial asthma who were treated between January 2024 and January 2025. Patients were divided into poor adherence [Morisky Medication Adherence Scale-8 (MMAS-8) score <6] and good adherence (MMAS-8 score 6–8) groups. Data on demographic characteristics, disease-related features, asthma control level, pulmonary function indices, and health-related quality of life (HRQoL) were collected and compared between the two groups.

**Results:**

Among the 268 children, 162 (60.4%) were in the poor adherence group and 106 (39.6%) in the good adherence group. The poor adherence group had a lower mean age (7.83 ± 1.72 vs. 8.47 ± 1.68 years), higher proportion of lower household income (10.49% vs. 4.72% with income <10,000 yuan), more frequent exacerbations in the past year (4.12 ± 1.24 vs. 2.53 ± 1.07), worse asthma control (30.25% vs. 10.38% uncontrolled), lower forced expiratory volume in 1 s (FEV₁: 75.34 ± 7.12% vs. 82.45 ± 6.34%), and lower total HRQoL score (69.36 ± 5.23 vs. 73.69 ± 5.12) (all *P* < 0.05). Multivariate analysis identified higher exacerbation frequency, uncontrolled asthma, hospitalization history, and family smoking as independent risk factors for poor adherence (all *P* < 0.05).

**Conclusion:**

Key modifiable barriers to inhalation therapy adherence in children with asthma at primary hospitals include frequent exacerbations, poor disease control, prior hospitalizations, and household smoking, highlighting the need for multifaceted interventions targeting these factors.

## 1 Introduction

Bronchial asthma remains one of the most prevalent chronic respiratory diseases affecting children worldwide, imposing a significant burden on individuals, families, and healthcare systems (1, 2). Characterized by chronic airway inflammation, variable airflow obstruction, and bronchial hyperresponsiveness, asthma in children often manifests as recurrent episodes of wheezing, breathlessness, chest tightness, and cough (1, 3). The cornerstone of effective long-term asthma management, as emphasized by international guidelines like the Global Initiative for Asthma (GINA), is consistent controller therapy, predominantly delivered via inhalation to maximize local efficacy and minimize systemic side effects (1, 4, 5). The 2022 GINA update reinforces that all asthma treatment should contain an inhaled corticosteroid (ICS), taken either regularly or, for mild asthma, as needed in conjunction with a symptom reliever (5). Inhaled corticosteroids (ICS), often combined with long-acting beta-agonists (LABA) such as budesonide/formoterol, are fundamental for suppressing inflammation, preventing exacerbations, and achieving optimal symptom control (1, 4).

Despite the proven efficacy of inhaled controller medications, achieving sustained disease control remains a major challenge, largely contingent on patients' long-term adherence to prescribed therapy (1, 5). Non-adherence is a complex, multifaceted problem recognized as a significant contributor to suboptimal asthma outcomes, including increased exacerbation frequency, emergency department visits, hospitalizations, reduced lung function, impaired quality of life, and higher healthcare costs (3, 5, 6). Pediatric asthma presents unique adherence challenges. Children are inherently dependent on caregivers for medication procurement, administration supervision, and understanding the necessity of daily therapy, even during asymptomatic periods (3). A 2022 qualitative study highlighted that during the COVID-19 pandemic, caregivers' health literacy and beliefs were significant internal factors affecting the implementation of asthma management plans, underscoring the critical role of the caregiver (7). Factors influencing adherence span diverse domains, including child-related aspects, caregiver/family factors, healthcare system factors, therapy-related issues, and disease-specific characteristics (3, 5, 8).

While substantial research exists on adherence barriers in asthma, a critical gap persists regarding the specific obstacles encountered within primary healthcare settings, particularly for pediatric populations in resource-limited contexts (9, 10). Primary hospitals often serve as the first and most frequent point of contact for the majority of children with asthma globally, especially in many regions, including China (11). These settings may face distinct challenges, such as potentially higher patient volumes, varying levels of provider training in pediatric asthma management and adherence counseling, diverse socioeconomic backgrounds of the patient population, and differing access to support resources compared to specialized tertiary centers (9, 11, 12). Understanding the context-specific barriers to long-term inhalation therapy adherence in children managed primarily at this level is therefore paramount.

Identifying the key modifiable obstacles hindering consistent medication use in this specific setting and population is essential for developing targeted, feasible, and effective interventions. A comprehensive framework, such as the SEIPS model, highlights that adherence is influenced by a complex system involving persons, tasks, tools, environment, and organizational factors (13). Current evidence suggests that interventions tailored to the local context and addressing the most prevalent barriers are more likely to succeed in improving adherence and, consequently, clinical outcomes (10, 14). However, a comprehensive analysis focusing explicitly on the multifaceted determinants of long-term adherence specifically among pediatric asthma patients receiving their primary care at non-tertiary hospitals is lacking. This study aims to address this critical knowledge gap. By systematically investigating the demographic, socioeconomic, clinical, familial, and potentially healthcare system-related factors associated with poor long-term adherence to inhalation therapy, this research aims to elucidate the predominant obstacles faced by children with asthma and their caregivers within the primary healthcare setting. The ultimate goal is to inform the design of practical strategies to enhance adherence and improve asthma control in this vulnerable population managed at the frontline of care.

## 2 Materials and methods

### 2.1 Ethics statement

This study was approved by the Institutional Review Board and Ethics Committee of our hospital. All procedures performed were in accordance with the ethical standards of the responsible committee on human experimentation and with the Helsinki Declaration. Written informed consent was obtained from the parents or legal guardians of all participating children after a detailed explanation of the study's purpose, procedures, potential risks, and benefits. The confidentiality and privacy of patients and their families were strictly maintained throughout the research process, with data anonymized before analysis.

### 2.2 Case selection

A retrospective analysis was conducted on the medical records of 268 pediatric asthma patients aged 4–16 years who visited our hospital between January 2024 and January 2025. The diagnosis of bronchial asthma was established according to the Chinese Guidelines for the Prevention and Treatment of Bronchial Asthma (2016 edition) (15) and the Global Initiative for Asthma (GINA 2019 update) (16). Diagnostic criteria included recurrent episodes of wheezing, shortness of breath, chest tightness, or cough, often worsening at night or in the early morning, and the presence of diffuse expiratory wheezing on auscultation. Included patients were those formally diagnosed with asthma and receiving standardized asthma treatment for more than three months, either at our hospital or elsewhere. Exclusion criteria encompassed patients currently participating in drug clinical trials or other interventional studies; those with less than three months of asthma treatment; individuals with underlying structural lung diseases such as bronchopulmonary dysplasia or bronchiolitis obliterans; patients with comorbid psychiatric disorders or severe complications like Tourette's syndrome; children or parents unable to complete the study requirements due to cognitive limitations; and cases where the child or parent voluntarily withdrew consent or refused to participate in the questionnaire survey.

### 2.3 Medication adherence assessment and grouping criteria

Medication adherence was evaluated using the Morisky Medication Adherence Scale-8 (MMAS-8), a widely validated tool for assessing adherence in chronic diseases. The Cronbach's alpha of this scale is 0.817 (17). For children under 12 years of age, the scale was completed by their parents or guardians; for children aged 12 years or older, self-reporting of their adherence was used. The MMAS-8 scoring system assigns points as follows: items 1–4 and 6–7 are scored 1 point for “No” and 0 for “Yes”; item 5 is reverse-scored; and item 8 uses a 5-point Likert scale (scored from 0 for “Every time” to 1 for “Never”). The total score ranges from 0 to 8, with scores categorized as follows: ≥8 indicating good adherence, 6 to <8 indicating medium adherence, and <6 indicating poor adherence. Based on these scores, patients were divided into two groups: the poor adherence group (MMAS-8 score <6, *n* = 202) and the good adherence group (MMAS-8 score 6–8, *n* = 114).

All enrolled children were prescribed a dry powder inhaler (Budesonide and Formoterol Fumarate Powder for Inhalation, manufactured by AstraZeneca, Wuxi, China; specification: 80 μg budesonide and 4.5 μg formoterol per inhalation), with a dosage of one inhalation twice daily.

### 2.4 Data collection

Clinical data for all study participants were systematically extracted from the electronic medical record system. The collected information encompassed the following key areas: (1) demographic characteristics, including age, gender, body mass index (BMI), annual household income level, and medical payment method; (2) clinical and disease-related features, such as family history of asthma, identified triggers, asthma severity classification based on the most severe exacerbation in the past year, temporal patterns of symptom exacerbation, duration of asthma, frequency of exacerbations in the past year, and history of asthma-related hospitalizations; and (3) caregiver and family background information, including parental education levels, family smoking status, custodial situation, and whether the child was the only child in the family. This comprehensive dataset was compiled to facilitate a thorough analysis of factors potentially associated with inhalation therapy adherence. Medical insurance type was categorized as “urban” or “rural” based on the national classification. Urban medical insurance included the Urban Employee Basic Medical Insurance and Urban Resident Basic Medical Insurance schemes, which offer higher reimbursement rates and broader access to specialist services. Rural insurance referred to the New Rural Cooperative Medical Scheme, typically associated with lower reimbursement and limited tertiary care access. Annual household income was self-reported in five brackets, but was not adjusted for family size or regional cost-of-living due to data limitations.

### 2.5 Evaluation criteria and tools

#### 2.5.1 Asthma control level

Asthma control was assessed using validated, age-specific questionnaires (TRACK for <5 years, C-ACT for 5–11 years, ACT for ≥12 years), in alignment with published guidelines. Age group assignment was strictly followed based on chronological age to prevent misclassification. This 5-item instrument assesses both impairment (frequency of respiratory symptoms, activity limitation, nocturnal awakenings over the past 4 weeks, and rescue medication use over the past 3 months) and risk domains pertinent to asthma control in young children with physician-diagnosed asthma or recurrent wheezing suggestive of asthma. A total score of ≤19 indicated uncontrolled asthma, 20–22 indicated partially controlled asthma, and ≥23 indicated well-controlled asthma. For children aged 5–11 years, asthma control was assessed using the Childhood Asthma Control Test (C-ACT), a 7-item questionnaire with 4 items answered by the child (with caregiver assistance for reading/comprehension if needed) and 3 items answered by the caregiver. The C-ACT scores were interpreted as follows: ≤19 indicated uncontrolled asthma, 20–22 indicated partially controlled asthma, and ≥23 indicated well-controlled asthma. For participants aged 12 years and above, the Asthma Control Test (ACT) was administered directly to the child. The ACT is a 5-item questionnaire rated on a 5-point scale reflecting the child's condition over the preceding 4 weeks, with scores interpreted as ≤19 (uncontrolled), 20–22 (partially controlled), and ≥23 (well controlled). The Chinese version of TRACK has demonstrated good internal consistency (Cronbach's *α* = 0.71) (18), while the C-ACT and ACT have established internal consistency reliability of 0.759 (19) and 0.861 (20), respectively. This standardized classification system enabled consistent assessment of asthma control levels across the entire pediatric population in our study.

#### 2.5.2 Pulmonary function testing

Spirometry was performed using an automatic pulmonary function analyzer provided by the German company Pari. Participants assumed an upright position with the head level and slightly extended to optimize airway patency. A nose clip was applied, and a disposable mouthpiece was used. After several tidal breaths, participants performed a maximal forced expiratory maneuver from total lung capacity (TLC), repeating the procedure three times with the best effort selected for analysis based on acceptability and reproducibility criteria. Key parameters measured included: Forced Vital Capacity (FVC), representing the maximal volume exhaled forcefully from TLC and serving as an indicator of larger airway expiratory resistance; Forced Expiratory Volume in the first second (FEV₁), a primary and highly reproducible index of airflow limitation and overall lung function impairment; Peak Expiratory Flow (PEF), the maximal flow achieved during forced exhalation, reflecting large airway patency and respiratory muscle strength, often reduced during acute asthma exacerbations; and the maximal flow rates at 25% (FEF₂₅%), 50% (FEF₅₀%), and 75% (FEF₇₅%) of expired FVC. FEF₂₅% primarily reflects early expiratory flow and larger airways, while FEF₅₀% and FEF₇₅% are sensitive indicators of flow in the mid-to-late expiration phase and are used collectively to assess small airway function and obstruction.

#### 2.5.3 Quality of life

Health-related quality of life (HRQoL) specific to asthma was evaluated using a modified version of the Asthma Quality of Life Questionnaire (AQLQ). The original AQLQ was established by Juniper et al. (21) demonstrates robust psychometric properties. For the current study population, considering regional factors, genetic background, and prevalent lifestyle habits, the instrument was adapted. The adapted questionnaire comprised 20 items categorized into four domains: Activity Limitation (6 items), Symptoms (7 items), Psychological Status (5 items), and Environmental Impact (2 items). The modified AQLQ showed high internal consistency (Cronbach's alpha = 0.901) and a high two-week reproducibility (ICC = 0.863) (22). Each item was rated on a 5-point scale where 1 indicated “Extremely bothered/impaired” and 5 indicated “Not bothered/impaired at all”. The total possible score ranged from 20 (indicating maximal impairment in quality of life) to 100 (indicating no impairment).

### 2.6 Statistical analysis

Data analysis was performed using SPSS version 29.0 statistical software (SPSS Inc., Chicago, IL, USA). Continuous variables that followed a normal distribution were expressed as mean ± standard deviation ($\bar{X}\pm s$) and compared between the good and poor compliance groups using the independent samples t-test. Categorical data were presented as frequencies and percentages [*n* (%)], and intergroup comparisons were conducted using the Chi-square (*χ*^2^) test. Spearman's rank correlation analysis was employed to assess the relationships between various factors and medication adherence. Variables showing significant associations in the univariate analyses were subsequently included in a multivariate logistic regression model (using the Enter method) to identify independent factors influencing poor inhalation therapy compliance. Results of the logistic regression were expressed as odds ratios (OR) with their corresponding 95% confidence intervals (CI). A two-tailed *p*-value of less than 0.05 was considered statistically significant for all tests. To address potential collinearity among severity-related predictors, multivariate logistic regression was employed after univariate screening. Collinearity diagnostics (VIF < 2) confirmed acceptable independence of included variables.

## 3 Results

### 3.1 Demographic and basic characteristics

The demographic and baseline characteristics of the children stratified by compliance group are presented in Table 1. Significant differences were observed between the groups. Children in the poor compliance group were significantly younger (7.83 ± 1.72 years) than those in the good compliance group (8.47 ± 1.68 years) (*t* = 3.029, *P* = 0.003). The distribution of annual household income level (*χ*^2^ = 10.725, *P* = 0.030) and medical payment method (*χ*^2^ = 12.149, *P* = 0.002) also differed significantly, with the poor compliance group having a higher proportion of lower-income families and reliance on out-of-pocket or rural medical insurance. Furthermore, the poor compliance group had a significantly higher proportion of families with members who smoked (48.77% vs. 30.19%, *χ*^2^ = 9.113, *P* = 0.003) and a lower proportion of only children (40.12% vs. 60.38%, *χ*^2^ = 10.529, *P* = 0.001). No significant differences were found for gender, BMI, or parental education level (Table 1).

**Table 1:** Demographic and basic characteristics of children with asthma by compliance group.

Parameters	Good compliance group (*n* = 106)	Poor compliance group (*n* = 162)	*t*/*χ*²	*P*
Gender, *n* (%)			0.373	0.541
Male	59 (55.66%)	84 (51.85%)		
Female	47 (44.34%)	78 (48.15%)		
Age (years)	8.47 ± 1.68	7.83 ± 1.72	3.029	0.003
BMI (kg/m^2^)	17.43 ± 1.45	17.24 ± 1.52	1.042	0.298
Annual Household Income Level, *n* (%)			10.725	0.030
<10,000 yuan	5 (4.72%)	17 (10.49%)		
10,000–80,000 yuan	32 (30.19%)	67 (41.36%)		
80,000–150,000 yuan	42 (39.62%)	57 (35.19%)		
150,000–300,000 yuan	21 (19.81%)	16 (9.88%)		
>300,000 yuan	6 (5.66%)	5 (3.09%)		
Medical Payment Method, *n* (%)			12.149	0.002
Out-of-pocket	10 (9.43%)	32 (19.75%)		
Rural Medical Insurance	32 (30.19%)	66 (40.74%)		
Urban Medical Insurance	64 (60.38%)	64 (39.51%)		
Father's Education Level, *n* (%)			5.291	0.152
Junior high school or below	21 (19.81%)	47 (29.01%)		
High school	32 (30.19%)	53 (32.72%)		
Associate degree	26 (24.53%)	36 (22.22%)		
Bachelor's degree or above	27 (25.47%)	26 (16.05%)		
Mother's Education Level, *n* (%)			6.575	0.087
Junior high school or below	19 (17.92%)	42 (25.93%)		
High school	34 (32.08%)	62 (38.27%)		
Associate degree	30 (28.30%)	38 (23.46%)		
Bachelor's degree or above	23 (21.70%)	20 (12.35%)		
Family Members Smoking, *n* (%)			9.113	0.003
Yes	32 (30.19%)	79 (48.77%)		
No	74 (69.81%)	83 (51.23%)		
Custodian Situation, *n* (%)			1.142	0.565
Single-parent family	11 (10.38%)	24 (14.81%)		
Dual-parent family	90 (84.91%)	130 (80.25%)		
Other	5 (4.72%)	8 (4.94%)		
Only Child, *n* (%)			10.529	0.001
Yes	64 (60.38%)	65 (40.12%)		
No	42 (39.62%)	97 (59.88%)		

### 3.2 Asthma control level

**Figure 1:**
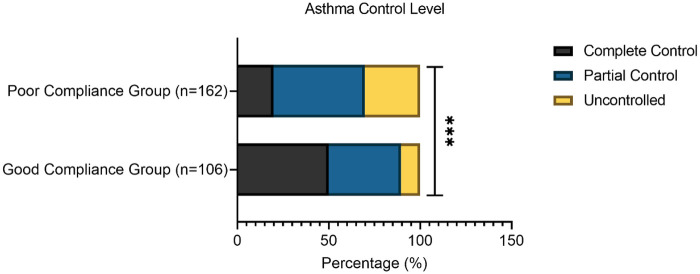
Asthma control level [n (%)].

Disease-related characteristics are summarized in Table 2. The poor compliance group had a significantly longer duration of asthma (4.18 ± 1.54 years vs. 3.47 ± 1.33 years, *t* = 3.911, *P* < 0.001) and a higher number of exacerbations in the past year (4.12 ± 1.24 vs. 2.53 ± 1.07, *t* = 10.887, *P* < 0.001). They also exhibited a more severe disease profile, with a higher proportion of severe acute exacerbations in the past year (29.63% vs. 10.38%, *χ*^2^ = 17.361, *P* < 0.001), a higher frequency of nighttime exacerbations (80.25% vs. 68.87%, *χ*^2^ = 4.516, *P* = 0.034), and a greater history of asthma-related hospitalization (40.12% vs. 19.81%, *χ*^2^ = 12.131, *P* < 0.001). Consequently, asthma control level was significantly worse in the poor compliance group (*χ*^2^ = 31.285, *P* < 0.001), with only 19.75% achieving complete control compared to 50.00% in the good compliance group, while 30.25% were uncontrolled compared to 10.38% (Figure 1).

**Table 2:** Disease-related general characteristics of children with asthma by compliance group.

Parameters	Good compliance group (*n* = 106)	Poor compliance group (*n* = 162)	*t*/χ²	*P*
Family History of Asthma, *n* (%)			0.719	0.396
Yes	27 (25.47%)	49 (30.25%)		
No	79 (74.53%)	113 (69.75%)		
Triggers, *n* (%)				
Sudden Temperature Drop	42 (39.62%)	81 (50.00%)	2.779	0.096
Exposure to Allergens	64 (60.38%)	113 (69.75%)	2.512	0.113
Respiratory Infections	85 (80.19%)	146 (90.12%)	5.314	0.021
Exercise	53 (50.00%)	97 (59.88%)	2.536	0.111
Strong Emotions	32 (30.19%)	65 (40.12%)	2.738	0.098
Classification of Most Severe Acute Exacerbation in Past Year, *n* (%)			17.361	< 0.001
Mild Exacerbation	53 (50.00%)	49 (30.25%)		
Moderate Exacerbation	42 (39.62%)	65 (40.12%)		
Severe Exacerbation	11 (10.38%)	48 (29.63%)		
Time Patterns of Asthma Exacerbations, *n* (%)				
Morning	21 (19.81%)	41 (25.31%)	1.089	0.297
Daytime	11 (10.38%)	24 (14.81%)	1.111	0.292
Nighttime	73 (68.87%)	130 (80.25%)	4.516	0.034
Irregular	32 (30.19%)	65 (40.12%)	2.738	0.098
Duration of Asthma (years)	3.47 ± 1.33	4.18 ± 1.54	3.911	< 0.001
Number of Exacerbations in Past Year (times)	2.53 ± 1.07	4.12 ± 1.24	10.887	< 0.001
History of Asthma-Related Hospitalization, *n* (%)			12.131	< 0.001
Yes	21 (19.81%)	65 (40.12%)		
No	85 (80.19%)	97 (59.88%)		

### 3.3 Pulmonary function indices

**Figure 2:**
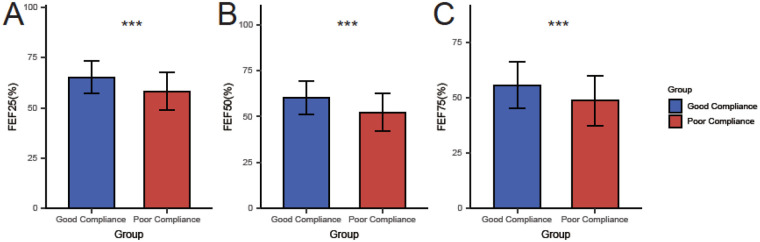
Comparison of large airway pulmonary function indices between the good and poor compliance groups (mean ± standard deviation). **(A)** Forced Vital Capacity (FVC, % predicted); **(B)** Forced Expiratory Volume in the first second (FEV₁, % predicted); **(C)** Peak Expiratory Flow (PEF, % predicted). ***P < 0.001 vs. Poor Compliance Group.

**Figure 3:**
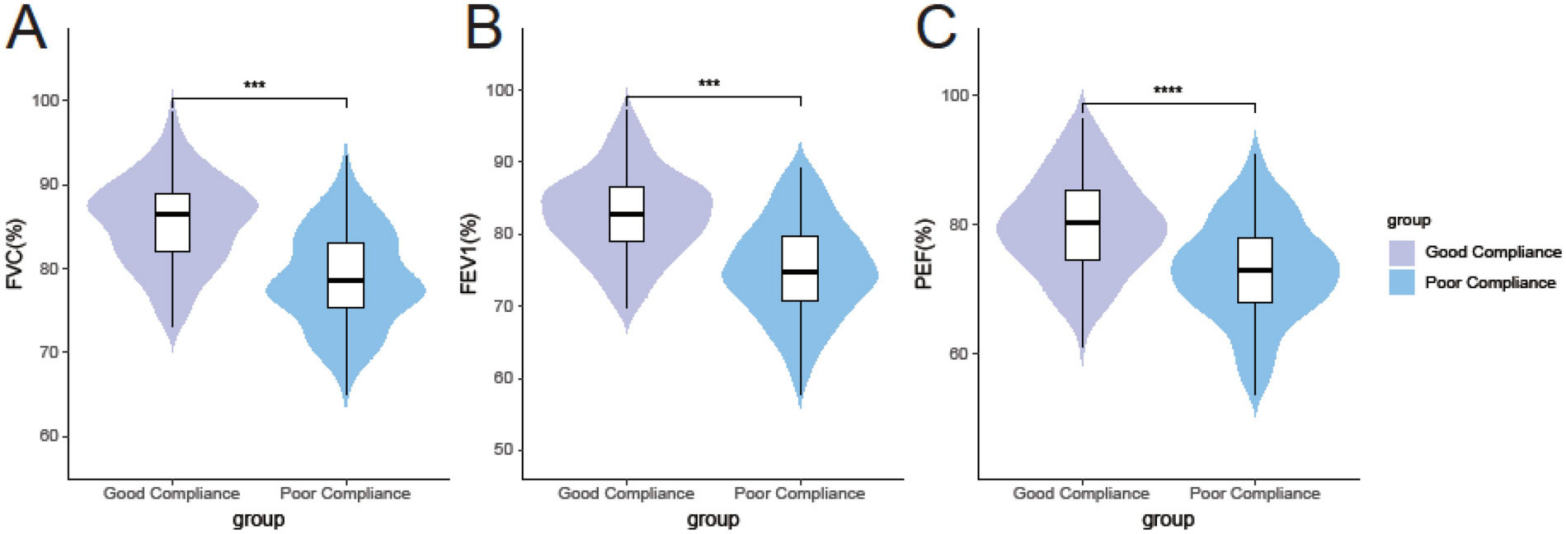
Comparison of small airway pulmonary function indices between the good and poor compliance groups (mean ± standard deviation). **(A)** Forced Expiratory Flow at 25% of FVC (FEF₂₅%, % predicted); **(B)** Forced Expiratory Flow at 50% of FVC (FEF₅₀%, % predicted); **(C)** Forced Expiratory Flow at 75% of FVC (FEF₇₅%, % predicted). ***P < 0.001 vs. Poor Compliance Group.

Analysis of pulmonary function revealed significantly better outcomes in the good compliance group for both large and small airway indices. As shown in Figure 2, large airway function parameters were higher in the good compliance group: FVC (85.23 ± 5.67% vs. 78.56 ± 6.45%, *t* = 8.674, *P* < 0.001), FEV₁ (82.45 ± 6.34% vs. 75.34 ± 7.12%, *t* = 8.336, *P* < 0.001), and PEF (80.12 ± 7.89% vs. 72.89 ± 8.23%, *t* = 7.143, *P* < 0.001). Similarly, small airway function indices were also superior in the good compliance group (Figure 3): FEF₂₅% (65.34 ± 8.23% vs. 58.23 ± 9.45%, *t* = 6.335, *P* < 0.001), FEF₅₀% (60.45 ± 9.12% vs. 52.34 ± 10.23%, *t* = 6.613, *P* < 0.001), and FEF₇₅% (55.67 ± 10.34% vs. 48.56 ± 11.12%, *t* = 5.257, *P* < 0.001).

### 3.4 Quality of life

Scores on the modified Asthma Quality of Life Questionnaire were significantly higher across all domains for the good compliance group compared to the poor compliance group (Table 3). The scores were as follows: Activity Limitation (21.43 ± 3.14 vs. 20.45 ± 3.56, *t* = 2.305, *P* = 0.022), Symptoms (26.67 ± 3.09 vs. 24.12 ± 3.12, *t* = 6.571, *P* < 0.001), Psychological Status (18.34 ± 2.08 vs. 17.56 ± 2.23, *t* = 2.870, *P* = 0.004), and Environmental Impact (7.67 ± 0.49 vs. 7.33 ± 0.51, *t* = 5.416, *P* < 0.001). The total quality of life score was consequently also significantly higher in the good compliance group (73.69 ± 5.12 vs. 69.36 ± 5.23, *t* = 6.674, *P* < 0.001).

**Table 3:** Scores of the modified asthma quality of life questionnaire (scores, $\bar{X}\pm s$).

Parameters	Good compliance group (*n* = 106)	Poor compliance group (*n* = 162)	*t*	*P*
Activity Limitation	21.43 ± 3.14	20.45 ± 3.56	2.305	0.022
Symptoms	26.67 ± 3.09	24.12 ± 3.12	6.571	< 0.001
Psychological	18.34 ± 2.08	17.56 ± 2.23	2.870	0.004
Environmental Impact	7.67 ± 0.49	7.33 ± 0.51	5.416	< 0.001
Total Score	73.69 ± 5.12	69.36 ± 5.23	6.674	< 0.001

### 3.5 Correlation analysis between variables and compliance

**Figure 4:**
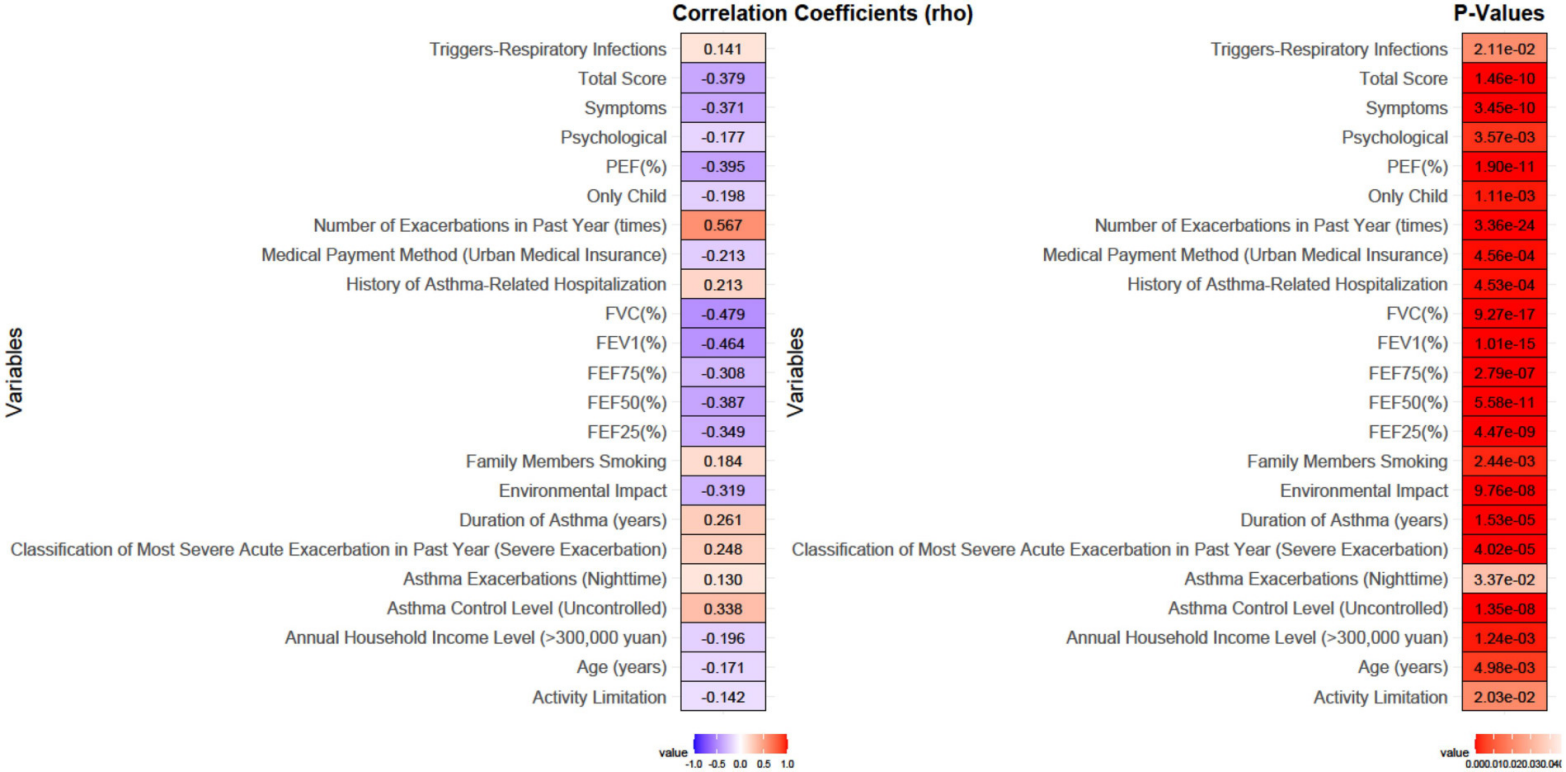
Correlation analysis between variables and poor compliance.

Spearman correlation analysis identified numerous factors significantly associated with poor compliance (Figure 4). Negative correlations were observed with age (rho = −0.171, *P* = 0.005), annual household income (rho = −0.196, *P* = 0.001), urban medical insurance (rho = −0.213, *P* < 0.001), being an only child (rho = −0.198, *P* = 0.001), and all pulmonary function and quality of life scores (e.g., FEV₁% rho = −0.464, Total QoL Score rho = −0.379; all *P* < 0.001). Positive correlations, indicating factors associated with poorer adherence, were found for family members smoking (rho = 0.184, *P* = 0.002), number of exacerbations in the past year (rho = 0.567, *P* < 0.001), history of hospitalization (rho = 0.213, *P* < 0.001), and uncontrolled asthma (rho = 0.338, *P* < 0.001).

### 3.6 Logistic regression analysis

Univariate logistic regression analysis confirmed that all variables listed in Table 4 were significant factors associated with poor compliance (all *P* < 0.05). The multivariate logistic regression model, presented in Table 5, identified several independent risk factors for poor adherence. These included a higher number of exacerbations in the past year (OR = 3.287, 95% CI: 2.402–4.498, *P* < 0.001), uncontrolled asthma (OR = 2.789, 95% CI: 1.886–4.129, *P* < 0.001), history of asthma-related hospitalization (OR = 2.665, 95% CI: 1.480–4.799, *P* = 0.001), and the presence of smoking family members (OR = 2.134, 95% CI: 1.257–3.621, *P* = 0.005). Protective factors included older age (OR = 0.823, 95% CI: 0.703–0.963, *P* = 0.015), higher household income (OR = 0.687, 95% CI: 0.522–0.837, *P* = 0.007), urban medical insurance (OR = 0.589, 95% CI: 0.406–0.855, *P* = 0.005), being an only child (OR = 0.473, 95% CI: 0.284–0.787, *P* = 0.004), higher FEV₁% (OR = 0.872, 95% CI: 0.830–0.916, *P* < 0.001), and a higher total quality of life score (OR = 0.841, 95% CI: 0.793–0.892, *P* < 0.001).

**Table 4:** Univariate logistic regression analysis of factors associated with poor inhalation therapy compliance.

Factors	Coefficient	Std Error	Wald Stat	P	OR	95%CI
Age (years)	−0.224	0.076	2.941	0.003	0.799	0.686–0.925
Annual Household Income Level (>300,000 yuan)	−0.429	0.137	3.128	0.002	0.651	0.495–0.848
Medical Payment Method (Urban Medical Insurance)	−0.624	0.185	3.366	<0.001	0.536	0.369–0.765
Family Members Smoking	0.789	0.264	2.993	0.003	2.201	1.321–3.720
Only Child	−0.822	0.255	3.219	0.001	0.440	0.265–0.722
Triggers-Respiratory Infections	0.813	0.359	2.266	0.023	2.254	1.120–4.616
Classification of Most Severe Acute Exacerbation in Past Year (Severe Exacerbation)	0.719	0.178	4.029	<0.001	2.052	1.457–2.938
Asthma Exacerbations (Nighttime)	0.608	0.288	2.111	0.035	1.836	1.044–3.239
Duration of Asthma (years)	0.339	0.091	3.720	<0.001	1.404	1.179–1.688
Number of Exacerbations in Past Year (times)	1.198	0.156	7.689	<0.001	3.314	2.486–4.588
History of Asthma-Related Hospitalization	0.998	0.292	3.421	<0.001	2.712	1.551–4.888
Asthma Control Level (Uncontrolled)	1.034	0.196	5.272	<0.001	2.812	1.935–4.182
FVC (%)	−0.178	0.026	6.933	<0.001	0.837	0.794–0.878
FEV_1_ (%)	−0.153	0.023	6.730	<0.001	0.858	0.819–0.895
PEF (%)	−0.113	0.019	6.036	<0.001	0.893	0.860–0.925
FEF_25_ (%)	−0.090	0.016	5.538	<0.001	0.914	0.884–0.942
FEF_50_ (%)	−0.084	0.015	5.746	<0.001	0.920	0.893–0.945
FEF_75_ (%)	−0.061	0.013	4.822	<0.001	0.941	0.917–0.964
Activity Limitation	−0.085	0.038	2.272	0.023	0.918	0.852–0.988
Symptoms	−0.269	0.047	5.722	<0.001	0.764	0.694–0.835
Psychological	−0.166	0.059	2.796	0.005	0.847	0.752–0.950
Environmental Impact	−1.363	0.278	4.899	<0.001	0.256	0.145–0.434
Total Score (Quality of Life)	−0.159	0.027	5.801	<0.001	0.853	0.807–0.898

**Table 5:** Multivariate logistic regression analysis of factors independently associated with poor inhalation therapy compliance.

Factors	Coefficient	Std error	Wald stat	*P*	OR	95%CI
Age (years)	−0.195	0.080	5.941	0.015	0.823	0.703–0.963
Annual Household Income Level (>300,000 yuan)	−0.375	0.140	7.174	0.007	0.687	0.522–0.837
Medical Payment Method (Urban Medical Insurance)	−0.529	0.190	7.750	0.005	0.589	0.406–0.855
Family Members Smoking	0.758	0.270	7.881	0.005	2.134	1.257–3.621
Only Child	−0.749	0.260	8.299	0.004	0.473	0.284–0.787
Number of Exacerbations in Past Year (times)	1.190	0.160	55.316	<0.001	3.287	2.402–4.498
History of Asthma-Related Hospitalization	0.980	0.300	10.671	0.001	2.665	1.480–4.799
Asthma Control Level (Uncontrolled)	1.026	0.200	26.317	<0.001	2.789	1.886–4.129
FEV_1_ (%)	−0.137	0.025	30.030	<0.001	0.872	0.830–0.916
Total Score (Quality of Life)	−0.173	0.030	33.255	<0.001	0.841	0.793–0.892

## 4 Discussion

This study aimed to elucidate the key obstacles affecting long-term adherence to inhalation therapy among children with bronchial asthma managed in primary hospital settings. By examining a range of demographic, socioeconomic, clinical, familial, and health-related quality of life factors, we identified several important associations with medication compliance. The findings highlight the multifaceted nature of adherence behavior and underscore the critical interplay between socioeconomic context, disease burden, family environment, and patient-centered outcomes in shaping long-term therapy persistence.

Beginning with demographic and socioeconomic characteristics, our analysis revealed that younger age was associated with poorer adherence. This aligns with observations by Baker (6), who noted that managing asthma in younger children poses unique challenges due to greater dependency on caregivers for daily medication administration. Furthermore, lower annual household income and reliance on out-of-pocket or rural medical insurance schemes, as opposed to urban medical insurance, were linked to reduced adherence. These findings are consistent with broader health disparities literature, which indicates that financial constraints can limit access to consistent medication supplies and follow-up care, thereby compromising adherence. The observed association between having smoking family members and poorer adherence may be explained by increased household stress, poorer indoor air quality, and potentially higher exacerbation frequency, which can disrupt care routines and diminish perceived treatment benefit (23, 24). Additionally, children who were not the only child in the family showed lower adherence, possibly reflecting divided caregiver attention and resources, as suggested in qualitative work by Goddard et al. (25), which emphasized the role of caregiver capacity in asthma management.

Regarding disease-related characteristics, children with poorer adherence exhibited a longer duration of asthma, a higher frequency of exacerbations in the past year, more severe acute exacerbations, a greater tendency for nighttime symptom worsening, and a higher likelihood of prior asthma-related hospitalization. These findings resonate with the studies by Alqarni et al. (26) indicating that poor adherence is both a cause and a consequence of uncontrolled disease. A cyclical relationship may exist wherein suboptimal adherence leads to more frequent and severe symptoms, which in turn can discourage consistent medication use due to frustration, hopelessness, or misunderstanding of controller therapy's preventive role (27, 28). The strong association between nighttime exacerbations and non-adherence underscores the impact of symptom burden on daily life and therapy routines, as nocturnal symptoms often disrupt sleep and reduce overall quality of life for both child and caregiver. The history of hospitalization may serve as a marker of disease severity but also reflect prior failures in management, potentially eroding caregiver confidence in treatment efficacy.

Pulmonary function assessments further supported the clinical relevance of adherence. Children in the good adherence group demonstrated better function across both large and small airway parameters. Impaired pulmonary function has been previously correlated with poor asthma control and adherence challenges [GINA report, as cited by Levy et al. (5)]. The observed differences in FEV₁ and small airway indices suggest that adherent children may experience less chronic airflow limitation, possibly due to consistent anti-inflammatory effects of inhaled corticosteroids. This reinforces the importance of adherence in preserving lung function over time, particularly during childhood when lung growth is ongoing. The link between adherence and small airway function is noteworthy, as these airways are increasingly recognized as key sites of inflammation in asthma, and their dysfunction may contribute to symptoms despite normal large airway parameters (29).

Health-related quality of life (HRQoL) scores were consistently higher across all domains among children with good adherence. This includes activity limitation, symptom burden, psychological status, and environmental impact. These results echo the work of Rönnebjerg et al. (30), who validated the importance of HRQoL assessment in asthma populations. Better adherence appears to facilitate improved day-to-day functioning and well-being, likely through reduced symptom frequency and severity. Conversely, poor HRQoL may demotivate adherence by reducing the perceived value of treatment, especially during asymptomatic periods. The psychological domain score, in particular, highlights the emotional toll of uncontrolled asthma, which can affect both the child's and the family's engagement with therapy (31).

The correlation and multivariate regression analyses synthesized these findings into a coherent model of adherence determinants. Independent risk factors for poor adherence included a higher number of exacerbations in the past year, uncontrolled asthma, a history of hospitalization, and presence of smoking family members. Protective factors included older age, higher household income, urban medical insurance, being an only child, better FEV₁%, and higher overall HRQoL score. These results are largely consistent with prior models of pediatric asthma adherence, such as those proposed by Baker (6) and He et al. (31), which emphasize the roles of socioeconomic support, family environment, and disease control. The strong independent effect of exacerbation frequency and asthma control level underscores that adherence is closely tied to illness perception and experience. Families who witness frequent exacerbations may become more vigilant, but those who feel treatment is ineffective may disengage. The protective effect of urban medical insurance likely relates to better financial coverage and access to specialist support, reducing practical barriers to refills and follow-up (32).

Several mechanisms may underlie these associations. Socioeconomic factors influence material resources and health literacy, affecting the ability to procure medications and understand long-term therapy needs. Family smoking not only directly exacerbates asthma but may also reflect broader health attitudes or stressors that impede consistent care. Disease-related factors like exacerbation frequency and nighttime symptoms impact both objective disease burden and subjective health beliefs, potentially triggering a negative feedback loop where poor control leads to poor adherence and vice versa. The quality of life and lung function findings suggest that adherence contributes to a positive cycle of better health outcomes and greater motivation to continue treatment.

This study has several limitations. Its retrospective design limits causal inference, and the single-center sample may affect generalizability to other primary care settings. Adherence was measured via self-report or parent-report, which is subject to recall and social desirability biases. Although we used validated tools, objective measures such as prescription refill data or electronic monitoring could strengthen future adherence assessments. Additionally, some potential confounders, such as detailed caregiver health beliefs or provider communication quality, were not captured. Household income was reported in absolute annual terms without normalization for household size or geographic cost-of-living variation, which may limit precision in assessing socioeconomic impact.

Future research should prospectively examine adherence barriers in diverse primary care contexts, incorporating mixed-methods approaches to explore caregiver and child perspectives. Intervention studies targeting modifiable factors—such as reducing household smoking, providing financial support, or simplifying treatment regimens—are needed to test strategies for improving adherence. Long-term follow-up would help clarify the temporal relationship between adherence, asthma control, and lung function growth. Ultimately, integrating multifaceted support into primary care pathways may help overcome the complex barriers to sustained inhalation therapy in children with asthma.

## 5 Conclusion

This study, set within a primary hospital context, identifies a cluster of interrelated and modifiable barriers—notably frequent exacerbations, suboptimal disease control, and household smoking—as critical obstacles to inhalation therapy adherence in children with asthma. These factors are closely linked to poorer lung function and reduced quality of life. The findings underscore that effective adherence-enhancing strategies for this setting must extend beyond mere education to address the clinical burden and family environment, advocating for integrated, multifaceted interventions tailored to the realities of primary care.

## Data Availability

The raw data supporting the conclusions of this article will be made available by the authors, without undue reservation.
